# Differentiation of benign and metastatic lymph nodes in soft tissue sarcoma

**DOI:** 10.1007/s10585-024-10273-7

**Published:** 2024-02-29

**Authors:** Anton Burkhard-Meier, Vindi Jurinovic, Luc M. Berclaz, Markus Albertsmeier, Hans Roland Dürr, Alexander Klein, Thomas Knösel, Dorit Di Gioia, Lena M. Unterrainer, Nina-Sophie Schmidt-Hegemann, Jens Ricke, Michael von Bergwelt-Baildon, Wolfgang G. Kunz, Lars H. Lindner

**Affiliations:** 1 Department of Medicine III, University Hospital, LMU Munich, Munich, Germany; 2 Institute for Medical Information Processing, Biometry, and Epidemiology, University Hospital, LMU Munich, Munich, Germany; 3 Department of General, Visceral and Transplantation Surgery, University Hospital, LMU Munich, Munich, Germany; 4 Department of Orthopedics and Trauma Surgery, University Hospital, LMU Munich, Munich, Germany; 5 Institute of Pathology, University Hospital, LMU Munich, Munich, Germany; 6 Department of Nuclear Medicine, University Hospital, LMU Munich, Munich, Germany; 7 Department of Radiation Oncology, University Hospital, LMU Munich, Munich, Germany; 8 Department of Radiology, University Hospital, LMU Munich, Munich, Germany

**Keywords:** Soft tissue sarcoma, Lymph node metastasis, Predictive parameters, Staging imaging

## Abstract

Lymph node metastasis (LNM) occurs in less than 5% of soft tissue sarcoma (STS) patients and indicates an aggressive course of disease. Suspicious lymph nodes (LN) in staging imaging are a frequent topic of discussion in multidisciplinary tumor boards. Predictive markers are needed to facilitate stratification and improve treatment of STS patients. In this study, 56 STS patients with radiologically suspicious and subsequently histologically examined LN were reviewed. Patients with benign (n = 26) and metastatic (n = 30) LN were analyzed with regard to clinical, laboratory and imaging parameters. Patients with LNM exhibited significantly larger short axis diameter (SAD) and long axis diameter (LAD) vs. patients with benign LN (median 22.5 vs. 14 mm, p < 0.001 and median 29.5 vs. 21 mm, p = 0.003, respectively). Furthermore, the presence of central necrosis and high maximal standardized uptake value (SUVmax) in FDG-PET-CT scans were significantly associated with LNM (60 vs. 11.5% of patients, p < 0.001 and median 8.59 vs. 3.96, p = 0.013, respectively). With systemic therapy, a slight median size regression over time was observed in both metastatic and benign LN. Serum LDH and CRP levels were significantly higher in patients with LNM (median 247 vs. 187.5U/L, p = 0.005 and 1.5 vs. 0.55 mg/dL, p = 0.039, respectively). This study shows significant associations between LNM and imaging features as well as laboratory parameters of STS patients. The largest SAD, SUVmax in FDG-PET-CT scan, the presence of central necrosis, and high serum LDH level are the most important parameters to distinguish benign from metastatic LNs.

## Introduction

Soft tissue sarcoma (STS) refers to a heterogeneous group of mesenchymal malignancies accounting for approximately 1% of all malignancies in adults [1]. Up to half of STS patients will develop metastatic disease, primarily affecting the lungs [2]. With survival rates of one to two years despite optimal treatment, prognosis remains dismal for advanced and metastatic STS patients [3, 4].

While hematogenous spread is common, lymph node metastases (LNM) are exceptionally rare with a reported incidence of 2.6–5.3% for STS [5–9]. Certain subtypes, such as synovial sarcoma, clear cell sarcoma, angiosarcoma, rhabdomyosarcoma and epithelioid sarcoma (acronym: SCARE) were found to be more likely to develop LNM, although latest reports exclude synovial sarcoma from this list [9]. Regional LNM, potentially indicating a disseminated disease and a high biological aggressiveness, is associated with poor survival. A comprehensive analysis of the American *National Cancer Database* demonstrated an overall survival of 28 months in STS patients with *pN1M0* disease varying by histology, approximating the prognosis of metastatic STS [6]. According to the current *American Joint Committee on Cancer* (AJCC) classification, any *N1M0* STS in the trunk and extremities is classified as stage IV, such as an M1 metastatic STS [10]. However, the significance of regional LNM remains controversial and patients with extremity STS and isolated LNM might have a better prognosis than the AJCC classification suggests [11].

Depending on pathological, radiological, and clinical assessment, STS patients are treated with either a potentially curative or palliative intent. In non-metastatic stages, complete surgical resection can be coupled with radio- and chemotherapy plus regional hyperthermia to reduce local and distant recurrence. For metastatic patients, palliative anthracycline-based chemotherapy remains the standard treatment [12, 13].

Frequently, STS patients present with suspicious lymph nodes (LN) in staging imaging, based on non-sarcoma specific criteria, such as pathological enlargement and the presence of a central necrosis [14, 15]. As nodal involvement indicates a more advanced stage, it might change the treatment strategy towards an extended surgical intervention or palliative therapy. Considering the strong impact on the therapeutic approach in these patients, it is important to carefully interpret suspicious LN in staging imaging and, if appropriate, to examine them histologically. To date, there are no accurate imaging or clinical criteria for the evaluation of LN involvement in STS. This study aimed to identify predictive parameters for LNM in STS patients in a German large-volume sarcoma center.

## Materials and methods

### Patient selection

Patients who underwent histological examination of suspicious LN in staging imaging with either histologically confirmed or excluded LNM of STS between 2006 and 2022 were included. Patients without a histological report or evaluable staging imaging of suspicious LN were excluded. The need for histological examination of LN was based on the following criteria: Short-axis diameter (SAD) ≥ 10 mm (according to response evaluation criteria in solid tumors [RECIST 1.1]) or significant asymmetry to the contralateral side defined as 2 × SAD of contralateral side, no fatty hilum, no calcification. Histological examination was either performed as a LN biopsy or in the context of a tumor resection. Clinical, imaging and outcomes data were extracted from our hospital database. Tumor and LN size were recorded from the imaging analysis and/or pathological report. Due to the anonymized nature of the study, informed consent was not required.

### Imaging analysis

Computed tomography (CT), magnetic resonance (MR) and positron emission tomography with 18-fluorodeoxyglucose (FDG-PET-CT) images were reviewed by one radiologist with subspecialty training in oncological imaging (WGK). The size of LN in terms of long axis diameter (LAD) and SAD as well as the number and presence of a central necrosis were evaluated. LN central necrosis is defined as a central area of low attenuation surrounded by an irregular periphery of enhancing tissue [15]. FDG uptake was quantified by calculating the maximal standardized uptake value (SUVmax) which indicates the degree of tumor metabolism. All CT images and the CT component of PET-CT exams were contrast-enhanced in the venous scan phase. For magnetic resonance imaging (MRI) scans, contrast-enhanced T1-weighted sequences were used. To evaluate signs of a possible benign course, T2-weighted images were additionally reviewed. Measures were performed on the most convenient CT or MRI sequences available. All scan protocols were standardized to ensure reproducibility. Follow-up images were reviewed for size (SAD and LAD) and occurrence of new LN according to the same technical conditions.

### Histopathology

Histopathology was performed according to standard procedures at the LMU Munich pathology department by a specialized sarcoma pathologist. The pathologists’ original report served as specification of tumor grade and LN histology.

### Statistics

Continuous variables were compared with the Mann–Whitney U test, and categorical variables with Fisher’s exact test. Receiver operating characteristic (ROC) curves were analyzed and compared for those variables that demonstrated a significant association with LN status. Optimal cut-off values from ROC curve analyses were determined by the highest Youden Index. Overall survival was analyzed using the Kaplan–Meier method and the log-rank test. The results with a p-value of less than 0.05 were considered statistically significant. Statistical analysis was performed using R software version 4.0.3 (R Foundation for Statistical Computing, Vienna, Austria).

## Results

### Patient cohort

The clinicopathologic characteristics of the study cohort are summarized in Table 1. A total of 56 STS patients with radiologically suspicious LN and either benign LN (group *B*, n = 26) or metastatic LN (group *M*, n = 30) evaluated by histological examination were included in our study. These two groups of patients were compared with regard to clinical parameters.

**Table 1:** Characteristics of patients with benign and metastatic lymph nodes

Factor	Benign LN (n = 26)	Metastatic LN (n = 30)	p value
Histological subtype			
UPS	7 (27%)	6 (20%)	0.180
Leiomyosarcoma	7 (27%)	2 (7%)	
Clear Cell Sarcoma	2 (8%)	3 (10%)	
Fibrosarcoma	2 (8%)	2 (7%)	
Dediff. Liposarcoma	2 (8%)	1 (3%)	
Synovial Sarcoma	2 (8%)	1 (3%)	
EHE	0 (0%)	3 (10%)	
Rhabdomyosarcoma	0 (0%)	3 (10%)	
Other (all STS ≤ 2 patients)	4 (15%)	9 (30%)	
Grading according to FNCLCC			
1	1 (4%)	3 (10%)	0.845
2	8 (31%)	7 (23%)	
3	13 (50%)	15 (50%)	
Not available/applicable	4 (15%)	5 (17%)	
Localization of primary tumor			
Extremities	11 (42%)	20 (67%)	0.238
Retroperitoneal/Abdominal	6 (23%)	1 (3%)	
Trunk	3 (12%)	2 (7%)	
Head/Neck	1 (4%)	1 (3%)	
Pleural/pulmonary	2 (8%)	3 (10%)	
Other	3 (12%)	3 (10%)	
Localization of suspicious LN			
Regional	18 (69%)	24 (80%)	0.375
Distant	8 (31%)	6 (20%)	
Date of occurrence of suspicious LN			
Synchronous	16 (62%)	18 (60%)	> 0.999
Metachronous	10 (38%)	12 (40%)	

*UPS* undifferentiated pleomorphic sarcoma, *EHE* Epithelioid hemangioendothelioma, *FNCLCC* Fédération Nationale des Centres de Lutte Contre le Cancer, *LN* Lymph nodes

Median age was similar in both groups (*B*: median 60 years, range 13–78 years vs. *M*: median 54 years, range 25–79 years), and a male predominance was observed in both groups (*B*: 62% and *M*: 70% of patients in each group). Undifferentiated pleomorphic sarcoma (UPS) represented the most common subtype in both groups (*B*: n = 7, 27%, *M*: n = 6, 20%) along with leiomyosarcoma in group *B* (n = 7, 27%), and followed by clear cell sarcoma in group *M* (n = 3, 10%). For patients with benign and metastatic LN, high tumor grade (G2/3; *B*: 81%, *M*: 73%), localization of the primary tumor on the extremities (*B*: 42%, *M*: 67%), regional (*B*: 69%, *M*: 80%) and synchronous occurrence (at initial diagnosis) of LN (*B*: 62%, *M*: 60%) were common characteristics. Primary tumor size did not differ significantly regarding the dignity of LN (*B*: median 9 cm, range 3.8–13.3 cm, *M*: median 7.1 cm, range 0.8–15 cm; p = 0.447).

Histopathological examination revealed LN *and* sarcoma tissue in 18 patients (60% of group *M*) and only sarcoma tissue in 12 patients (40%). For patients with benign LN, LN tissue without specification (n = 9, 34.6% of group *B*), reactive LN (n = 7, 23.3%), granulomatous inflammation (n = 4, 15.4%), sinus histiocytosis/anthracosis (n = 3, 11.5%) and fibrosclerotic tissue (n = 3, 11.5%) were reported.

Median overall survival (OS) from initial suspicion of LNM was 8.74 years for patients with benign LN and 3.16 years for patients with metastatic LN (p = 0.006).

### Imaging characteristics

**Fig. 1:**
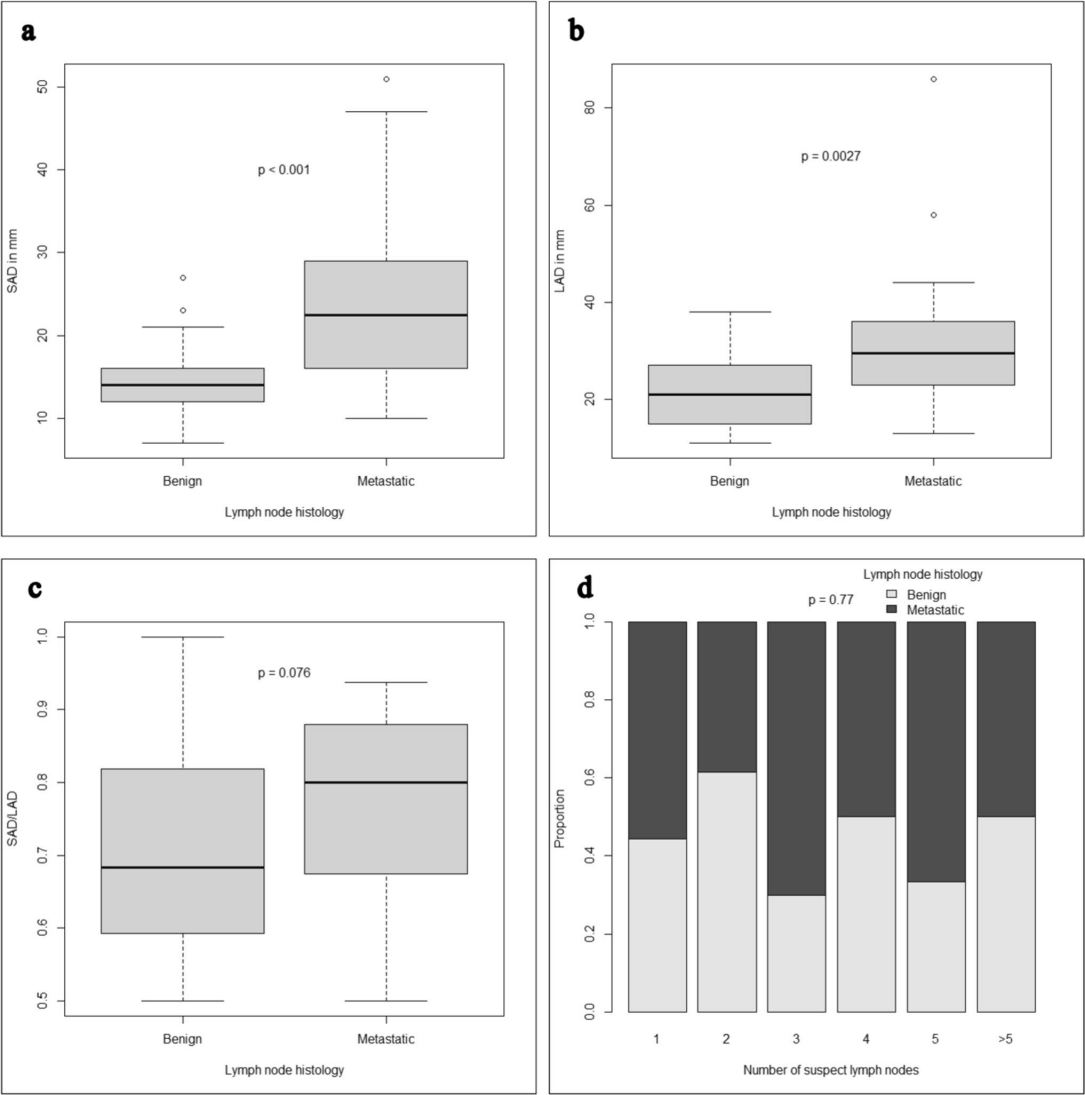
Imaging characteristics: Size in terms of short axis diameter (SAD) and long axis diameter (LAD) in mm (**a** and **b**), SAD/LAD ratio (**c**) and number of suspicious lymph nodes (**d**) in benign (n = 26) and metastatic (n = 30) lymph nodes in soft tissue sarcoma patients

The following imaging modalities demonstrating the suspicious LN were analyzed: CT in 31 patients, PET-CT in 14 patients and MRI in 11 patients. In staging imaging, the number of suspicious LN did not significantly differ in metastatic and benign patients (p = 0.770). However, a significantly larger SAD (*B*: median 14 mm, range 7-27 mm vs. *M*: median 22.5 mm, range 10–51 mm, p < 0.001) and LAD (*B*: median 21 mm, range 11-38 mm vs. *M*: median 29.5 mm, range 13–86 mm, p = 0.003) were observed in metastatic LN. The SAD/LAD ratio tended to be higher for metastatic LN (*B*: median 0.68, range 0.5–1 vs. *M*: median 0.8, range 0.5–0.94, p = 0.076) (Fig. 1).

**Fig. 2:**
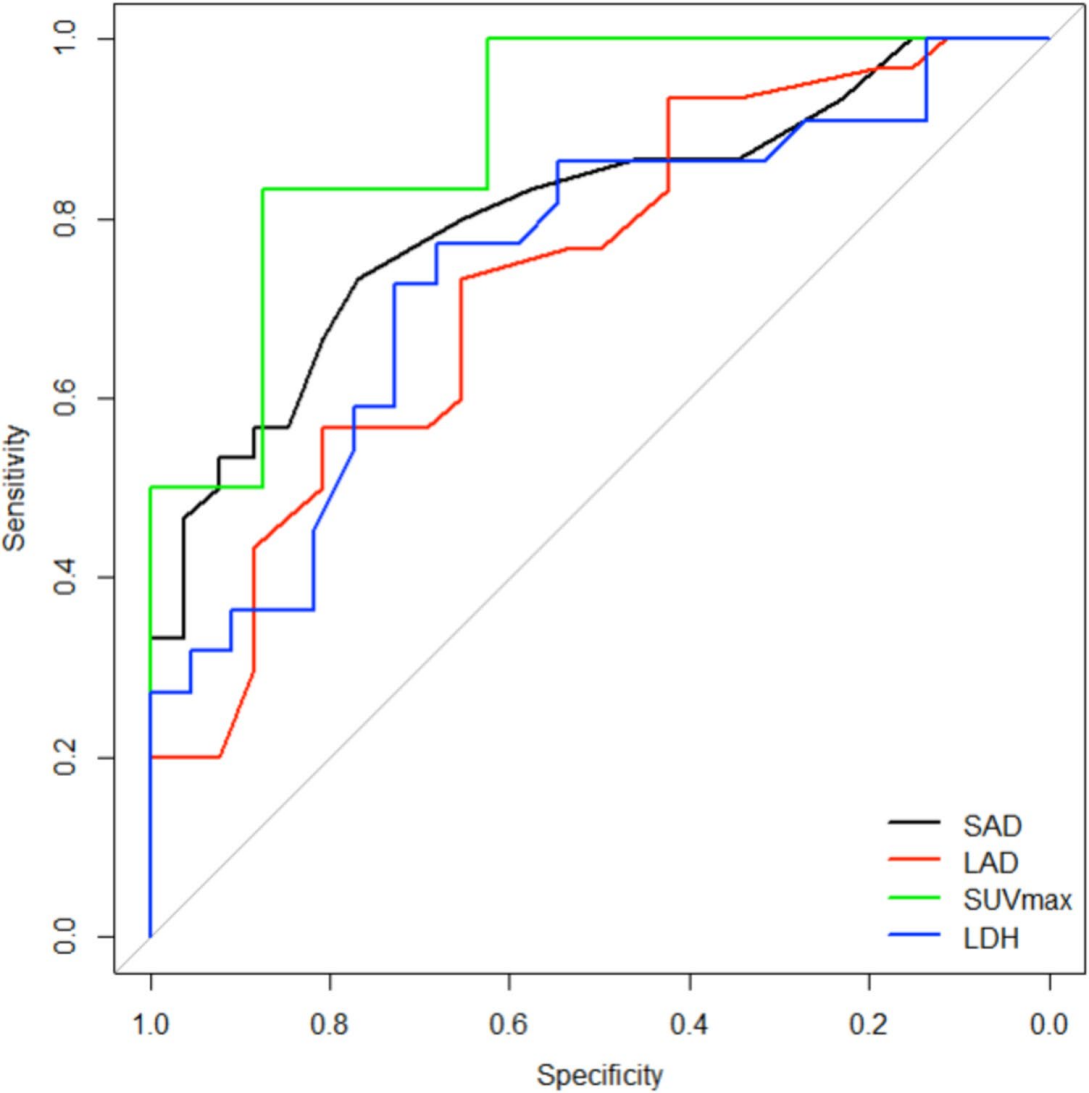
Receiver operating characteristic curve for short axis diameter (SAD), long axis diameter (LAD), maximal standardized uptake value (SUVmax) and lactate dehydrogenase (LDH)

ROC analysis revealed an area under the curve (AUC) of 0.81 for SAD and an AUC of 0.73 for LAD. Sensitivity and specificity were 73.3%, 76.9% for SAD with a cut-off value of 17 mm and 73.3%, 65.4% for LAD with a cut-off value of 24 mm (Fig. 2).

**Fig. 3:**
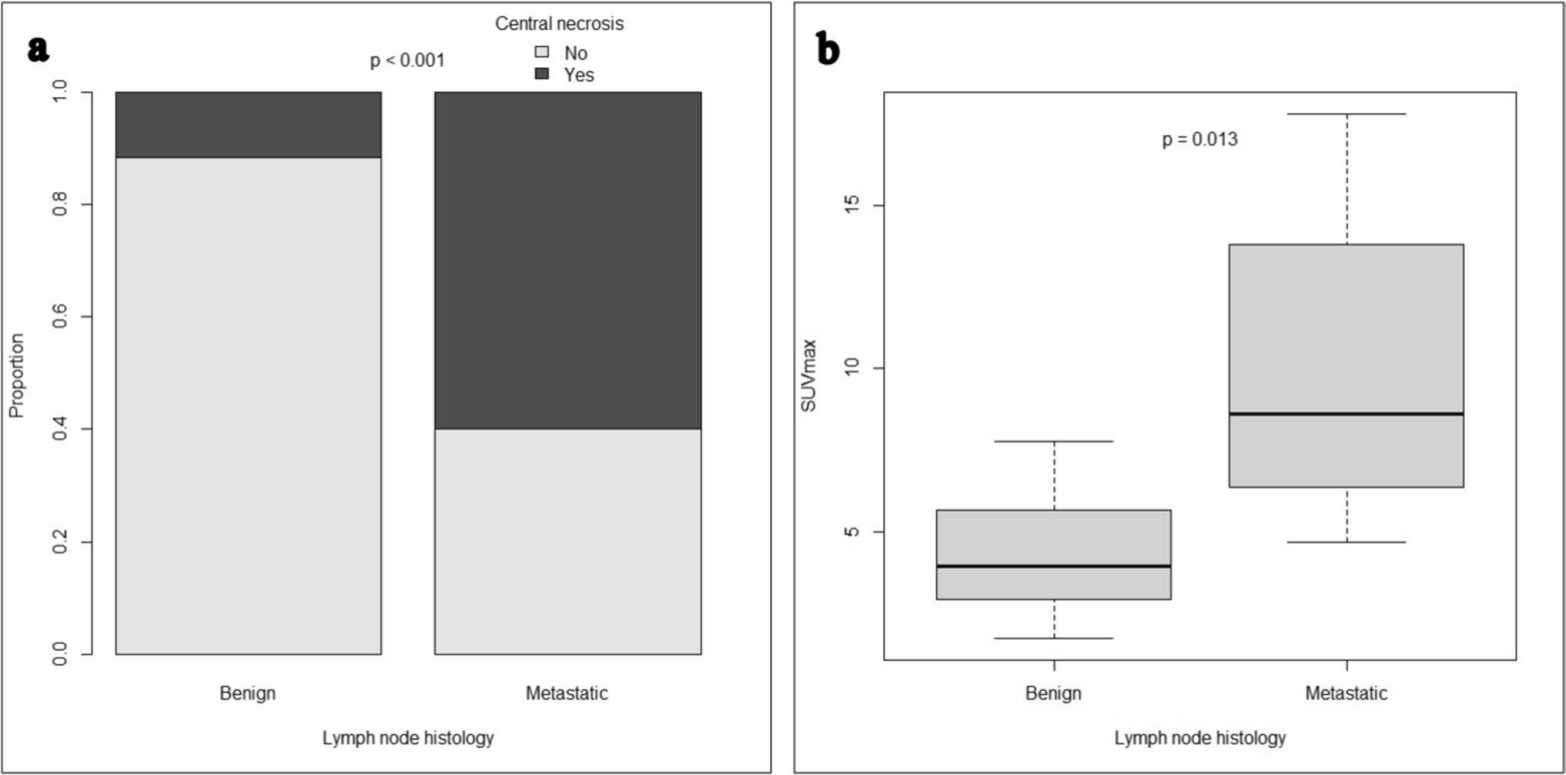
Imaging features: Presence of central necrosis (**a**) and maximal standardized uptake value (SUVmax) in FDG-PET-CT (**b**) in benign (n = 26 and n = 8, respectively) and metastatic (n = 30 and n = 6, respectively) lymph nodes in soft tissue sarcoma patients

Central necrosis was detected in 60% (n = 18) of patients with metastatic LN, compared with only 11.5% (n = 3) of patients with benign LN (p < 0.001). PET-CT imaging at initial suspicion was performed in 25% of patients (*B*: n = 8, *M*: n = 6). SUVmax was significantly higher for patients with metastatic LN (*B*: median 3.96, range 1.74–7.78 vs. *M*: median 8.59, range 4.68–17.8, p = 0.013) (Fig. 3). ROC analysis could determine an AUC of 0.9 with a sensitivity and specificity of 83.3%, 87.5% for a cut-off value of 6.38 (Fig. 2).

### Blood parameters

In our patient cohort, routine blood parameters were available in 45 patients (80.4% of all patients, *B*: n = 22, *M*: n = 23). The date of included blood sampling ranged from ten days before to 53 days after the first radiological suspicion. Serum lactate dehydrogenase (LDH) levels were significantly higher in patients with metastatic LN (*B*: median 187.5U/L, range 122-279U/L vs. *M*: median 247U/L, range 163-468U/L, p = 0.005). C-reactive protein (CRP) levels also proved to be significantly higher in these patients (*B*: median 0.55 mg/dL, range 0.1–5.8 mg/dL vs. *M*: median 1.5 mg/dL, range 0.1–24.4 mg/dL, p = 0.039), whereas leukocytes did not differ between the two groups (*B*: median 7.57G/L, range 3.4–12.6G/L vs. *M*: median 8.7G/L, range 3.6–21.4G/L, p = 0.218). AUC for LDH was 0.75 with a sensitivity and specificity of 72.7%, 68.2% for a cut-off value of 226 U/L (Fig. 2).

### Follow-up imaging

**Fig. 4:**
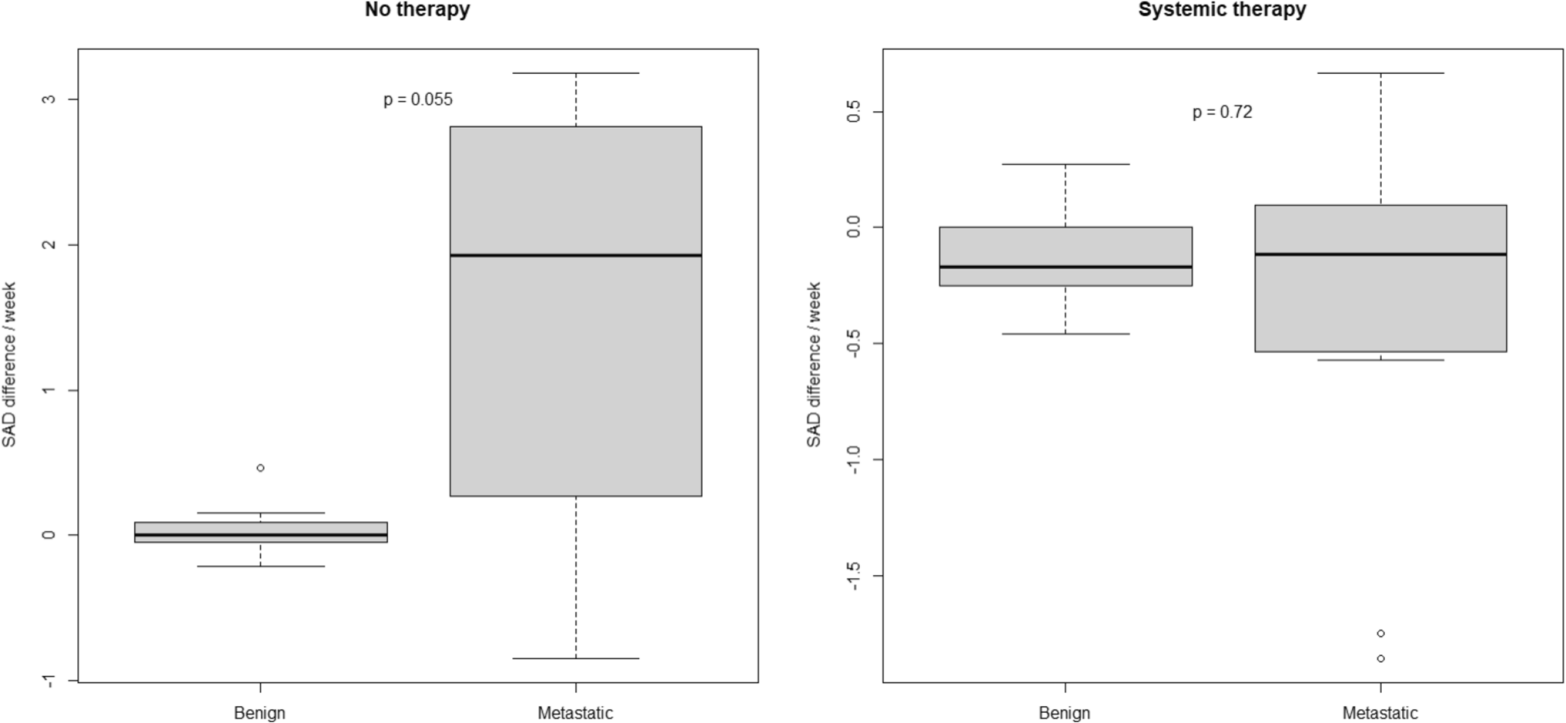
Follow-up imaging: Difference in short axis diameter of lymph nodes in mm per week under systemic therapy (benign: n = 13, metastatic: n = 12) or without therapy (benign: n = 7, metastatic: n = 8)

For 41 patients, follow-up imaging was available in a range of 1.7–76 weeks (median 9.8 weeks). CT, PET-CT and MRI images were analyzed in 28, 7 and 6 patients, respectively. Of these patients, 61% (n = 25) received a systemic therapy between initial and follow-up imaging (*B*: n = 13, *M*: n = 12), and 36.6% (*B*: n = 7, *M*: n = 8) did not receive a therapy in this time interval. One patient with metastatic LN was treated with radiotherapy. As the time interval between imagings varied between patients, size difference per week was calculated. Without therapy, benign LN presented a median SAD difference of 0.0 mm/week (range − 0.21 to 0.47) while metastatic LN showed a median growth of 1.92 mm/week (range − 0.85 to 3.18). Under systemic therapy, a slight SAD regression of LN was equally observed in benign and metastatic patients (*B*: median − 0.17 mm/week, range − 0.46 to 0.27 mm/week vs. *M*: median − 0.11 mm/week, range − 1.86 to 0.67 mm/week) (Fig. 4). New suspicious LN in the follow-up imaging were only observed in two metastatic patients (one with and one without therapy).

**Fig. 5:**
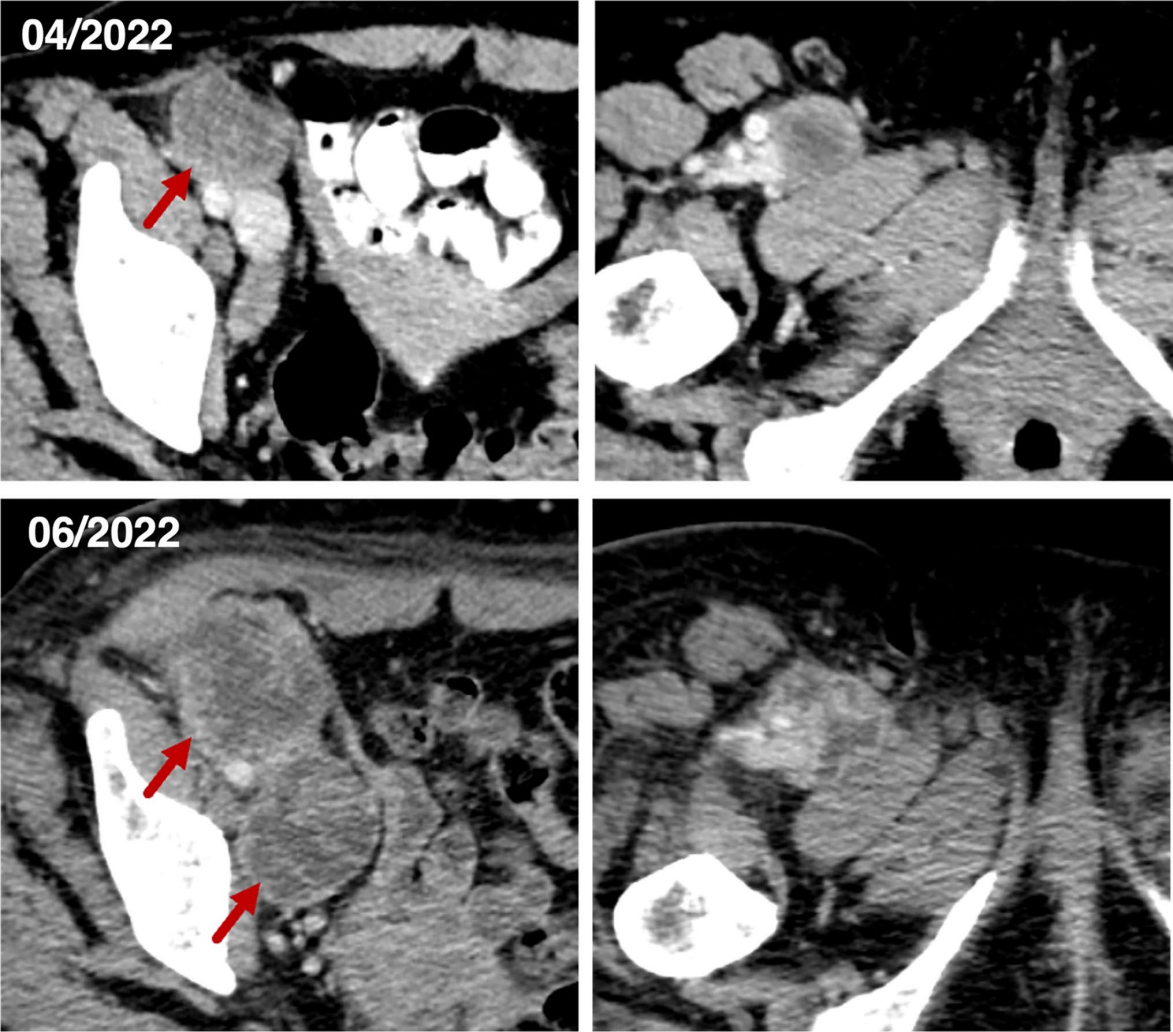
CT staging imaging of a 59-year-old female patient with an undifferentiated pleomorphic sarcoma in the right thigh region and metastasis to iliac and inguinal lymph nodes at baseline (04/2022) and after two cycles with doxorubicin/ifosfamide (06/2022). A central necrosis was identified in the detected metastatic lymph nodes at both dates. Besides a size progression, new suspicious lymph nodes occurred in follow-up imaging

**Fig. 6:**
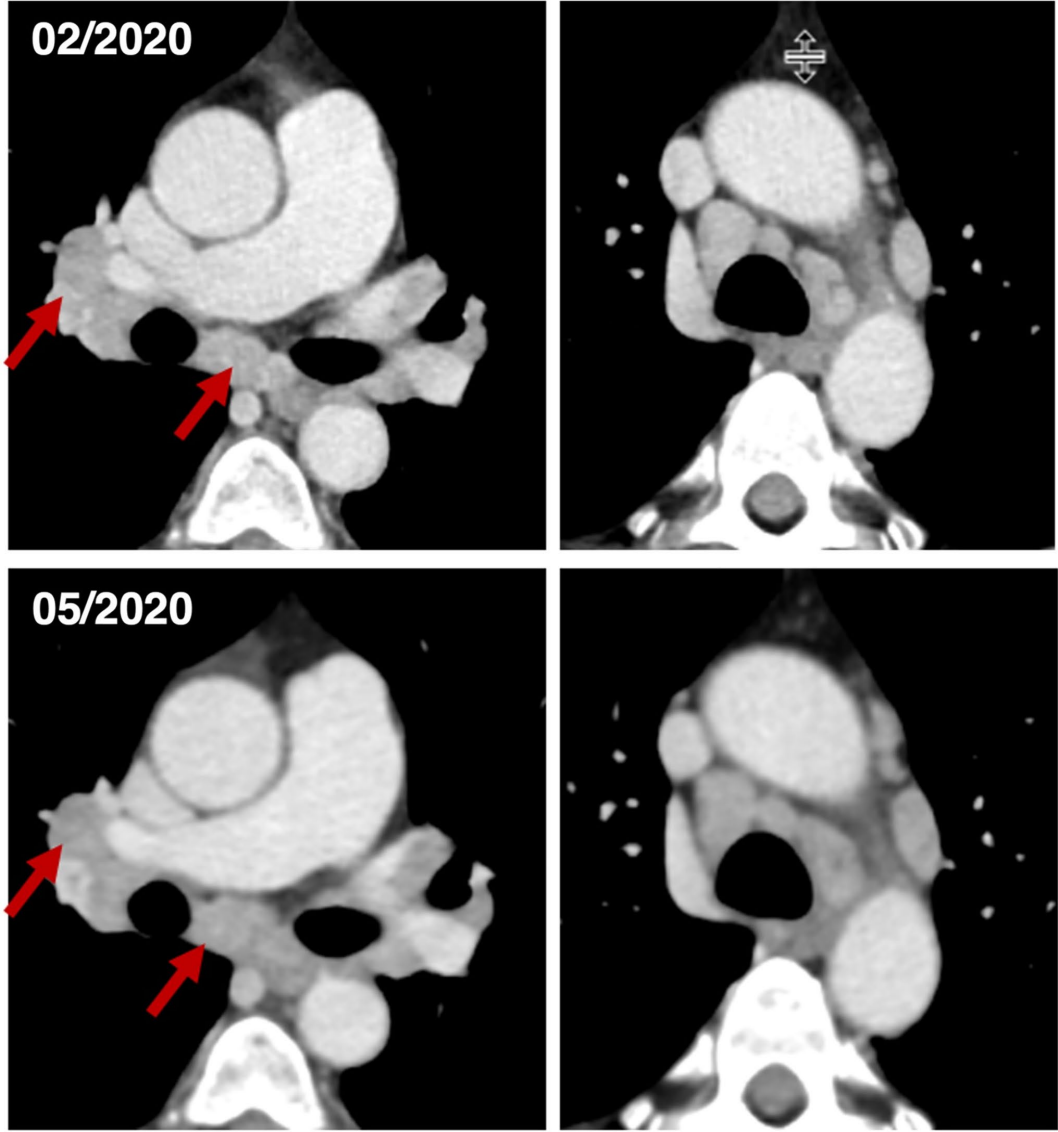
CT follow-up staging of a 65-year-old female patient three months after curative resection of a uterine leiomyosarcoma (02/2020). Another follow-up staging three months later (05/2020) showed no relevant changes. Suspicious bihilar lymph nodes proved to be epithelioid cell granuloma without necrosis compatible with a sarcoid-like reaction

Follow-up imaging of two exemplary patients is illustrated in Figs. 5 and 6.

## Discussion

Due to its rarity, LNM in STS remains insufficiently characterized. It is associated with a severe course of disease, and certain subtypes, such as clear cell sarcoma, angiosarcoma, rhabdomyosarcoma and epithelioid sarcoma seem more likely to present with LNM. There is limited evidence for the clinical management of LNM, including the value of lymphadenectomy and sentinel LN biopsy (SLNB) [16].

To our knowledge, this is the first study to compare STS patients with suspicious LN in imaging and histological confirmation (*pN1*) or exclusion of LNM (*pN0*). To date, no imaging criteria for LNM in STS have been established. Our results demonstrate a significant association of LN size in terms of LAD and SAD with LNM. ROC analysis suggests an SAD of 17 mm and an LAD of 24 mm as possible predictive cut-off values for LNM.

Enlargement of LN commonly leads to suspicion, but nodal size criteria vary depending on location and entity [17]. Furthermore, in other entities, such as non-small cell lung cancer (NSCLC) and gastric cancer, a lack of correlation between LN size and metastatic infiltration has been reported [18, 19]. Considering the low sample size and the heterogeneity of our cohort, LN in different locations were reviewed together as being a limitation of this study. The SAD/LAD ratio tended to be higher in our metastatic patients (p = 0.076), which is in line with previous literature across entities [20, 21]. Based on the observation that malignant LN lose their oval shape and become more circular, a high SAD/LAD ratio has been recognized as a potential criterion for LNM across different LN regions. Historically, central necrosis has been considered as the best criterion in CT scans for cervical LNM with an accuracy of nearly 100% [15]. In our cohort, we could confirm a strong association with central necrosis and LNM. However, 11.5% (n = 3) of benign patients did also present with a central necrosis while 40% (n = 12) of metastatic patients did not. It should be noted that one of the benign patients received a neoadjuvant chemotherapy after initial imaging and before histological examination. In this case, a complete regression might have led to the benign histological finding.

While CT and MRI serve as the standard-of-care in staging of STS, FDG-PET-CT is individually used to exclude distant metastasis by including metabolic characteristics [13]. In previous studies a high sensitivity and specificity of 90–100% for FDG-PET-CT in detecting LNM in bone and soft tissue sarcoma has been demonstrated. However, the positive predictive value varied and tended to be rather low due to FDG uptake in inflammatory tissue [22, 23]. In our cohort, only 14 patients received an FDG-PET-CT, and SUVmax was significantly higher in LNM patients with a predictive cut-off value of 6.38 after ROC analysis. The large range of SUVmax in benign and malignant LN might suggest a weak predictive value of this modality. Regarding the small and heterogenous group of patients the absolute cut-off value of this study should be interpreted with caution and needs to be validated in prospective studies.

When histological examination is not performed, LN size changes under systemic therapy might provide further information about their dignity. In our study, we analyzed the follow-up imaging of 41 patients. As expected, metastatic LN showed size progression without therapy while benign LN remained stable. Under systemic therapy, benign and metastatic LN behaved similarly and showed a slight median size regression. This might be a result of the immunosuppressive effect of chemotherapy, affecting both malignant and reactive tissue.

To find more predictive parameters beyond imaging, blood serum parameters including leukocyte counts, CRP and LDH around the time of initial suspicion were reviewed. In our cohort, LDH and CRP were significantly higher in LNM patients, which aligned with previous reports about high LDH and CRP values in an advanced tumor stage [24, 25]. Due to the volatile nature of CRP and leukocyte counts, and the possible impact of concomitant infections or other stress situations, these results must be interpreted with caution.

Our study does not necessarily provide valid information about the subtypes with the highest incidence of LNM due to sole inclusion of patients with histologically examined LN. To some extent, patients with STS at high risk of LNM might have directly received a systemic therapy without histological examination of LN. UPS constituted the most common subtype of our patients with benign and metastatic LN (27 and 20% respectively). In addition to its heterogeneous and aggressive profile, the immunogenic nature of UPS might be a reason for a high incidence of suspicious LN in this subtype [26]. However, rare subtypes at high risk for LNM, such as rhabdomyosarcoma, clear cell sarcoma and epithelioid sarcoma were also part of our cohort. As implied by our results, male sex has been associated with LNM in extremity STS in previous literature [27].

Limitations of our study include the small size of our cohort and the retrospective design. Typically for STS studies, the patient cohort is very heterogeneous including many different subtypes and locations. Furthermore, a high selection bias must be assumed as only patients with biopsy were included.

## Conclusion

To our knowledge, this study is the first attempt to predict LN status for patients with soft tissue sarcoma. We found that a large LN size in terms of short axis diameter, the presence of central necrosis and a high SUVmax are strong imaging features associated with LN positivity for patients with soft tissue sarcoma. High serum LDH values could also serve as a predictive parameter of LNM. LN size regression with systemic therapy should not routinely be interpreted as a response to therapy and evidence of malignancy. As the ranges of all parameters overlap in benign and metastatic LN, biopsy should always be performed in case of uncertainty.

## Data Availability

The data that support the findings of this study are not openly available due to reasons of sensitivity and are available from the corresponding author upon reasonable request.
